# Seamless integration of image and molecular analysis for spatial transcriptomics workflows

**DOI:** 10.1186/s12864-020-06832-3

**Published:** 2020-07-16

**Authors:** Joseph Bergenstråhle, Ludvig Larsson, Joakim Lundeberg

**Affiliations:** Science for Life Laboratory, Department of Gene Technology, KTH Royal Institute of Technology, Tomtebodavägen 23, SE-171 65 Solna, Sweden

**Keywords:** Spatial transcriptomics, Transcriptomics, Genomics, Software, Visualization, Image processing, Data analysis, R-package, 3D

## Abstract

**Background:**

Recent advancements in in situ gene expression technologies constitute a new and rapidly evolving field of transcriptomics. With the recent launch of the 10x Genomics Visium platform, such methods have started to become widely adopted. The experimental protocol is conducted on individual tissue sections collected from a larger tissue sample. The two-dimensional nature of this data requires multiple consecutive sections to be collected from the sample in order to construct a comprehensive three-dimensional map of the tissue. However, there is currently no software available that lets the user process the images, align stacked experiments, and finally visualize them together in 3D to create a holistic view of the tissue.

**Results:**

We have developed an R package named STUtility that takes 10x Genomics Visium data as input and provides features to perform standardized data transformations, alignment of multiple tissue sections, regional annotation, and visualizations of the combined data in a 3D model framework.

**Conclusions:**

STUtility lets the user process, analyze and visualize multiple samples of spatially resolved RNA sequencing and image data from the 10x Genomics Visium platform. The package builds on the Seurat framework and uses familiar APIs and well-proven analysis methods. An introduction to the software package is available at https://ludvigla.github.io/STUtility_web_site/.

## Background

Gene expression analysis has become a standard tool for the study of gene regulation, cellular state and biological function in both bulk and single cell samples. However, the transcriptional profiles of individual cells are influenced by their localization in the tissue. As such, important information for the complete understanding of the biological underpinnings reflected in the transcriptional state requires simultaneous knowledge about morphological context [1, 2]. Recent technological advances have enabled this spatial information to be simultaneously obtained together with quantitative transcript measurements.

Spatial Transcriptomics (ST) is a method [1–3] that can be used to profile the transcriptome of tissues spatially and is today widely accessible using the 10x Genomics Visium platform. Overall, the spatial methods are quickly gaining traction among researchers, and lately several computational software packages have been released with support for spatial analyses [4–7]. However, there is currently no software package for ST data that lets the user process the images, align stacked experiments, and finally visualize them together in 3D to create a holistic view of the tissue.

## Implementation

Here, we present STUtility, an R package that conveniently enables the user to perform these tasks. STUtility can be used for normalization, identification of spatial expression patterns alignment of consecutive stacked tissue images and visualizations. STUtility builds on the Seurat framework and uses familiar APIs and well-proven analysis methods. STUtility uses RNA count and image data as input. Specifically, it is created with compatibility for using 10x Genomics Space Ranger output, which is a set of analysis pipelines provided by 10x Genomics that process raw Visium RNA-seq and image data (https://support.10xgenomics.com/spatial-gene-expression/software). All analysis, image processing and visualization in STUtility is conducted within R.

## Results and discussion

The poly A targeted whole transcriptomics spatial methods are continuously improving the boundaries of cellular resolution. Nevertheless, the commercially available alternatives have not yet reached single-cell level, and, in their current format, typically capture transcripts from multiple cells in each spatial measurement spot. Although the data is derived from a mixture of cells, its characteristics are similar to those of single-cell RNA-seq (scRNA-seq) [8]. One of the main challenges in both ST and scRNA-seq, from a data analysis perspective, is the sparsity of the data (capture efficiency). Moreover, differences in tissue permeabilization, reverse transcription efficiency and other sources of technical noise may elevate the challenges. Hence, data transformation and normalization prior to various downstream analysis steps plays a major role in the ability to correctly characterize meaningful biological regional differences in transcriptional profiles [9]. Technical variation is further increased by the fact that comprehensive tissue models usually consist of multiple tissue sections, which may have been sequenced at different points in time, using different capture arrays, and with slightly different conditions. Various data transformation strategies have been proposed, and current state-of-the-art normalization methods for scRNA-seq data are based on statistical modeling. For example, SCTransform is a method available in the Seurat v3 R package [8] that uses a regularized negative binomial regression model and manages to retain the biological heterogeneity within the tissue sections while still being able to remove much of the technical variation. Seurat is widely used within the field, displays good performance in benchmarking studies [10], is frequently updated and works well with larger data sets [11]. Accordingly, we have chosen to build our visualization tool on top of the Seurat framework.

**Fig. 1:**
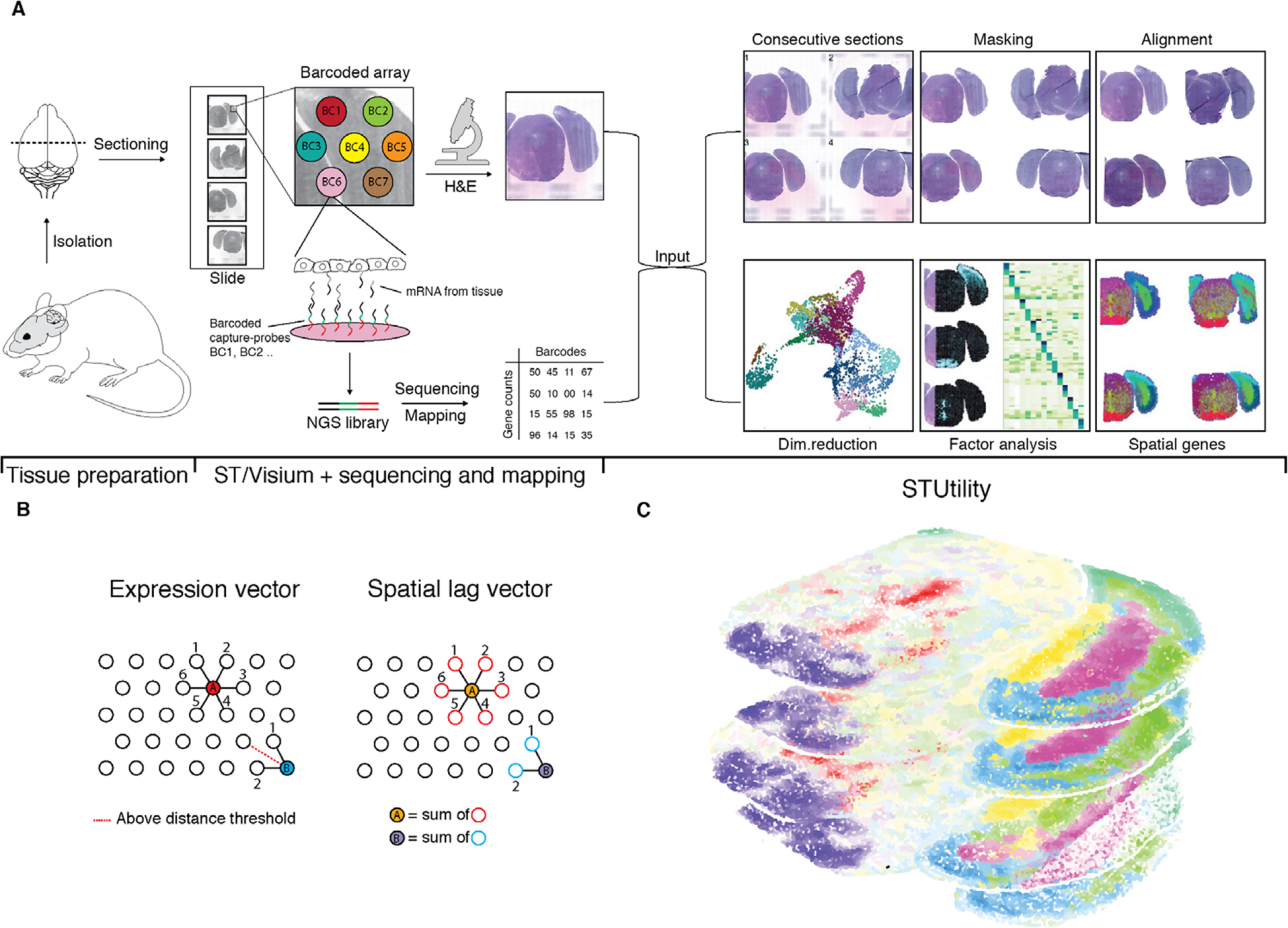
Schematic overview of the procedure, from tissue collection to final visualization of the data analysis results. **a** Thin tissue sections are placed on the ST/Visium array. Barcoded capture-probes store spatial information which is added to the captured transcript prior to sequencing. Imaging data is obtained by microscopy of stained tissue sections. The sequencing data is used as input to demultiplexing and transcript quantification pipelines. The count data together with the image data are used as inputs to STUtility. Image processing (including masking and alignment), and all further data analysis (e.g. dimensionality reduction, factor analysis, identification of spatially correlated genes) is conducted within R. **b** Spatial autocorrelation. Two vectors are defined: (i) the original expression vector for each gene and each capture-spot and (ii) the Spatial lag expression vector, which for each capture-spot and gene takes the summed expression of up to six neighbors. Spatial autocorrelation is defined as the Pearson correlation between the two vectors (i) and (ii) with the rationale that genes with spatial structure will display a higher correlation to their neighbors. **c** The aligned images can be visualized in a turntable 3D model within R in which a combination of features can be visualized. Here, the NMF factors of the tissue are shown in the HSV color scale

As an introduction to STUtility, we have established a website (https://ludvigla.github.io/STUtility_web_site/) that outlines its functionality and provides examples of the available features. Here, we give a short demonstration of the capabilities by using publicly available 10x Genomics Visium and in-house generated ST data. The complete STUtility workflow is conducted within R (Fig. 1). The input to STUtility consists of count files and bright field image data from Hematoxylin and Eosin (H&E) stained tissue sections, and all Seurat functions for handling and transforming the data can be utilized. On top of this, STUtility adds multiple features for spatial analysis, image processing and visualization.

The image processing procedure includes (i) masking the tissue to remove the array background from the image, (ii) automatic alignment of multiple samples, or (iii) manual alignment with an interactive Shiny R application.

**Fig. 2:**
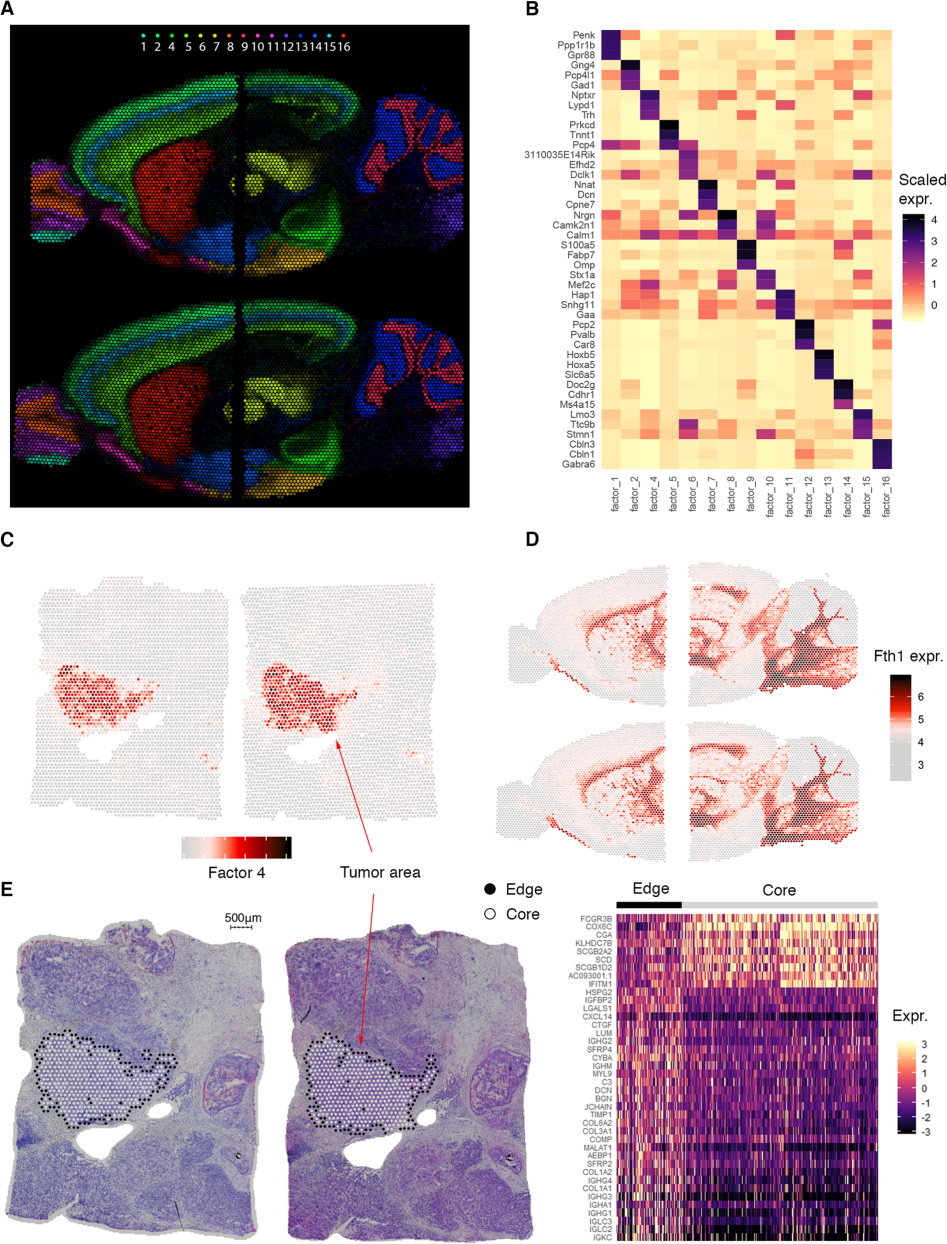
Spatial analysis of sagittal mouse brain and human breast cancer samples. **a** NMF identifies multiple spatially distinct factors within the mouse brain (4 separate tissue sections) that are visualized in the HSV color scale. **b** Visualization of driver genes of some of the NMF factors seen in (**a**). **c** NMF factor with clear histological relevance corresponding to a tumor area within the breast cancer samples. **d** Example of a top-ranking gene, *Fth1*, according to the proposed spatial autocorrelation metric performed on two adjacent sections to increase the robustness of the analysis. **e** NMF factors were clustered in Seurat, and capture-spots neighboring to one of the tumor clusters were automatically extracted by STUtility (left) for a differential expression analysis between core and tumor edge. The core and tumor edge display significant differences in expression of various immunoglobulin and Extracellular Matrix (ECM) related genes (right)

The masking procedure converts a low-resolution representation of the HE images into “superpixels”, followed by k-means clustering into two groups to segment out the areas located inside or outside of the tissue (Additional file 1). For the automatic alignment strategy, a reference image is selected and the remaining images are then aligned to the reference using an iterative closest point algorithm (ICP) [12] (Additional file 2: Figure S1). The ICP algorithm finds a transformation matrix that maps coordinates from each sample to the reference and can handle rigid transformations, i.e. offsets along the x and y axis (translations), rotations and reflections. If the automatic alignment fails, the transformation matrix can be found by manually aligning the images in a user interface (Additional file 2: Figure S2). The user can choose to utilize the masked and aligned images in all subsequent STUtility plotting functions. Moreover, the aligned images can be stacked to create a turntable 3D model of the tissue, which e.g. can be used to visualize gradually shifting changes in gene expression (Additional file 2: Figure S3). This 3D model is constructed by running a simple cell segmentation on each aligned tissue section based on intensity differences compared to the non-nuclei compartments of the cell to produce 2D point patterns. Although not offering a precise cell segmentation, this approach is able to sufficiently capture the overall morphological structure of the tissue in a time efficient manner (Additional file 2: Figure S4). The complete 3D stack is then created by assigning a z-coordinate to each 2D point pattern of nuclei from each tissue section, whereupon various strategies for visualization can applied. For example, STUtility provides a multiple feature view using the HSV color scale (Fig. 2a, Additional file 2: Figure S5) to visualize the spatial localization of non-overlapping features as exemplified in mouse hippocampus. Each selected feature is assigned a unique color defined by splitting the hue parameter into even breaks. Next, the feature values are rescaled to values between 0 and 1 and encoded in the V-channel. Then, for each spot, the feature with the highest value is selected to color the spot based on the feature associated hue and value. A value of zero gives the capture area a black color regardless of feature, whereas higher values increase the intensity of the corresponding feature color. This allows for simultaneous visualization of multiple non-overlapping features, giving a clear representation of, for example, distinct factor-based transcriptomic profiles of interest.

Added analysis functionality includes a Non-negative Matrix Factorization (NMF) [13] to decompose ST data into a lower dimensional space (Fig. 2a-c), a test to rank genes by degree of spatial autocorrelation (Fig. 2d) and a method for identification and extraction of neighboring capture-spots to a region of interest within a connected spatial network (Figs. 1b and 2e). The ranking method makes use of connection networks (or neighboring graphs) where each capture-spot (node) is connected to neighboring spots located within or close to the minimum center to center distance (i.e. 100 μm for Visium arrays) to form a local neighborhood. Neighborhood networks can be defined across multiple sections simultaneously and then used to compute the spatial lag for each gene, defined as the summed expression of that gene across the connected spots. Each gene is then ranked by the Pearson correlation between the lag vector and the original expression vector in decreasing order. The same connection network can be used for extracting neighboring capture-spots for a region of interest. This method can for example be used on cancer tissue as a tool to automatically define the border surrounding a tumor region to study tumor heterogeneity represented by unique transcriptome signatures within the tumor core compared to tumors along the tumor front (edge) (Fig. 2e).

STUtility also includes a manual annotation tool based on the Shiny R framework. The manual annotation tool allows the user to interactively select and label individual or sets of capture areas. These labels are saved within the S4 object and can be used, for example, during supervised differential gene expression (DGE) between regions of interest.

To demonstrate the STUtility workflow for ST and 10x Visium data, we performed analyses of multiple tissues from mouse and humans (as exemplified below). An identical workflow was used for all sample types to demonstrate the usability of the general workflow: NMF was used to uncover low-dimensional structure of tissues based on the high-dimensional count data and the spatial autocorrelation test was used to find genes with clear spatial patterns. Various visualization strategies available in STUtility after the image processing steps where then utilized to explore the results.

In *mouse brain* samples, multiple distinct factors could be extracted with well-defined spatial patterns (Fig. 2a-b), and the top ranked genes from the spatial autocorrelation test displayed clear spatial dependencies in their expression (Additional file 2: Figure S6). In the *breast cancer* tissue samples, clearly separated areas of tumor cells can be defined from the H&E images by histopathology. However, the morphological features alone do not show the remarkable heterogeneity of cellular states present within the tumor. Using the NMF based factor analysis in STUtility, this diversity can be comprehensibly visualized, and driver genes for the different tumor areas can be identified to strengthen the mechanistic interpretation (Additional file 2: Figure S7). The driver genes reveal a striking difference in ongoing cellular dynamics at the time of sampling. For example, one of the tumor areas factors (factor_3) is driven by immune response related genes (CPB1, HLA-B/C, PSMB8, IL6ST). Another factor instead displays a unique expression profile of markers previously shown to be dysregulated in various tumor types (e.g. *MGP, S100, TFF3*) [14–16]. Factor_7 on the other hand is concentrated to a small region between tumor areas with a strong signature for the presence and regulation of T and B cells (CCL19, TRBC2, CD52, MS4A1, TRAC), possibly forming a tertiary lymphoid structure [17]. Exploring further, observations from the outer region (leading edge) of the tumor area with elevated immune activity found earlier can be automatically extracted from the neighborhood network constructed by STUtility. Differential expression analysis (DEA) displays high expression of immunoglobulin genes in the outer (tumor leading edge) region compared to the inner core region of the tumor (Fig. 2e). Clusters can favorably be visualized using the split view functionality (Additional file 2: Figure S8). In the *lymph node* sample, the NMF based factor analysis reveals several distinct spatial patterns; factor_1 associated with an anti-viral response within a smaller sub-area of the lymph node and factor_2 and factor_4 showing clear signatures of T cell and Plasma cell activity (Additional file 2: Figure S9). Finally, *rheumatoid arthritis* samples were used to demonstrate the 3D application to a sample set consisting of multiple consecutive ST sections. The user can contract or expand the space between the sections, in order to either create a dense point cloud reflecting actual distance or introduce spacing for easier identification of feature transitions between sections (Additional file 2: Figure S10). Overall, the spatially resolved data, analyzed using STUtility, is able to present a landscape view about the ongoing cellular states of the tissue.

## Conclusions

Most of the spatial transcriptomics methods to date are based on using thin tissue sections, approximately the thickness of a cell, resulting in a 2D picture of the transcriptional state at a certain point in time at the precise location of sampling within the whole tissue. In order to achieve a comprehensive profile, multiple sections can be consecutively sectioned and aligned. The aligned images can then also be used to create a 3D model for visualization purposes. However, there is currently no tool available that performs this alignment or visualization in an easy and automatic fashion. Moreover, manual alignment quickly becomes cumbersome for large image stacks.

Here, we presented STUtility, a tool that deliver a complete workflow for spatial transcriptomics data, from sequencing and image data processing to the creation of a final 3D model of the tissue. The tool handles data from previous ST arrays [1–3, 18] and 10x Genomics Visium arrays and is delivered as an open and accessible R package.

## Availability and requirements

STUtility is compatible with R version > = 3.6 and has the following dependencies: akima (> = 0.6), imager (> = 0.41), magick (> = 2.0), Matrix (> = 1.2), Morpho (> = 2.7), Rvcg (> = 0.18), Seurat (> = 3.0.0), spatstat (> = 1.61), viridis (> = 0.5), data.table (> = 1.12.2), ggplot2 (> = 3.2), ggiraph (> = 0.6), imagerExtra (> = 1.3.2), NMLM (> = 0.4.3), raster (> = 3.0), reshape2 (> = 1.4.3), scales (> = 1.0), shinyjs (> = 1.0), SingleCellExperiment (> = 1.2). STUtility is available at https://github.com/jbergenstrahle/STUtility.

## Methods

### Data input and transformation

The same workflow was used for all samples: The count and image data were used as input to STUtility, and filtering was performed to remove lowly abundant genes and capture-spots with too little information by thresholding on the raw count values. The *SCTransform* function in Seurat was used to normalize the data.

### Factor analysis and clustering

Non-negative Matrix Factorization (NMF) was used to decompose the normalized data into factor activities. The NMF method can be accessed through the *RunNMF* function in STUtility which is created based on a method described earlier [13], using Independent Component Analysis (ICA) as initialization method and non-negative transformed Pearson residuals as input data. In short, the NMF method decomposes the gene expression matrix A into two non-negative matrices A = WH, where W is the feature loadings matrix (features x factors) and H is a matrix representing the data in a low dimensional space (factors x spots). Gene drivers were selected for each factor by extracting the top ranked features in the feature loadings matrix W. The low dimensional representation was then used as input to the Seurat functions *FindNeighbors* and *FindClusters*.

### Spatial autocorrelation

Specific genes demonstrating spatial patterns were obtained with a ranking method for spatial autocorrelation, where a connection network for each capture-spot is created based on the distance between the spot and its neighbors. The threshold for classifying a spot as a “neighbor” was set to 150 μm. The networks were used to compute the spatial lag for each gene, defined as the summed expression of that gene across the spots classified as neighbors to the capture-spot under consideration. The Pearson correlation between the spatial lag vector and the normalized count vector was then used to determine the overall spatial correlation across the tissue section. To demonstrate the validity of using the Pearson correlation for this usage, a code notebook demonstration is provided in Additional file 6.

### Differential gene expression

DEA between the outer and the inner region of the tumor was conducted with the *FindMarkers* function provided in the Seurat R package, after detecting the border capture-spots with the *RegionNeighbours* function in STUtility. The *FindMarkers* function was run with default parameters, which imply a non-parametric Wilcoxon rank sum test.

### Image processing – masking

Prior to visualization of results, image processing was performed. First, the H&E images were downscaled to a width of 400 pixels with retained aspect ratios, do demonstrate this ability which is used to reduce memory demands. A threshold was put on the downscaled images using Otsu’s method as implemented in the threshold function of the imager R package. Isotropic blurring was applied to the images, and a Simple Linear Iterative Clustering (SLIC) algorithm was used to convert the images into superpixels [19], i.e. a clustering of pixels based on color similarity in the image plane. Finally, k-means (*kmeans* function in the stats package, with k = 2) clustering was used to segment the images into two regions: inside/outside tissue. A visual demonstration of the masking process is shown in Additional file 1.

### Image processing – alignment

Alignment of the tissue sections was performed by ICP as implemented in the R package Morpho, using the first image as reference. As input to the ICP algorithm, we used the pixel coordinates of the edges of the tissue defined during the masking procedure. First, the tissue edges are detected from the previously defined image masks by computing an image gradient (*imgradient* in imager R package). Next, the tissue edge pixel coordinates are extracted from the images and leveraged into the ICP method, computed for each pair of target samples and reference. The ICP method returns a transformation matrix for each sample that can be used to map coordinates between the sample and reference. The image alignment is then performed using the *imwarp* function (imager R package) using the transformation matrix defined by ICP as the mapping function. To achieve a smooth transformation of the images, the direction was set to “backward” with a “cubic” interpolation.

For specific parameters used during the filtering and factor analysis steps, see the individual RMarkdown files for each sample (Additional files 3, 4 and 5).

## Supplementary information

**Additional file 1:** A step by step description of the masking procedure.

**Additional file 2:** Supplementary figure S1-S10.

**Additional file 3:** RMarkdown which includes the analysis of the 10x Genomics public data sets.

**Additional file 4:** RMarkdown which includes the 3D view of rheumatoid arthritis data.

**Additional file 5:** RMarkdown on how to use the RegionNeighbours function.

**Additional file 6:** HTML notebook demonstrating spatial autocorrelation.

## Data Availability

STUtility is available at https://github.com/jbergenstrahle/STUtility. The source code of STUtility is freely available under the MIT license. RMarkdown documents with the code to produce the results and figures are found in Additional files 3, 4 and 5. A tutorial and online documentation is provided at https://ludvigla.github.io/STUtility_web_site/. ST mouse brain samples shown for demonstration purposes of image alignment are included in the R package as example data. These samples are processed according to the Spatial Transcriptomics method described earlier [1]. For the RA samples, H&E images and count matrices were downloaded from https://www.spatialresearch.org/resources-published-datasets/ licensed under the Creative Commons Attribution license. 10x Visium data were downloaded from the publicly available datasets on the 10x Genomics website (https://www.10xgenomics.com/resources/datasets/) licensed under the Creative Commons Attribution license. Specifically, the following data sets were used: Mouse Brain Serial Sections (Sagittal-Posterior library T1T2-F3, H3 and Sagittal-Anterior library T1T2-G3, A4). Human Breast Cancer (Block A Section 1 and 2, library T1T2-F10 and T1T2-H10) Human Lymph Node (library T1T2-E3).

## Abbreviations

DGE: Differential gene expression
HSV: Hue, Saturation, and Value
ICA: Independent Component Analysis
ICP: Iterative closest point
NMF: Non-negative matrix factorization
scRNA-seq: Single-cell RNA sequencing
SLIC: Simple Linear Iterative Clustering
ST: Spatial transcriptomics
