# Supplementary material for: Seamless integration of image and molecular analysis for spatial transcriptomics workflows
Source: BMC Genomics. 2020 Jul 16;21:482. doi: 10.1186/s12864-020-06832-3 (PMC7386244; doi:10.1186/s12864-020-06832-3)
Supplement: Supplementary file 1 — Additional file 1: A step by step description of the masking procedure. [file 12864_2020_6832_MOESM1_ESM.docx]

**Masking**

**Input:**

Raw H&E image downscaled to a width of 400 pixels


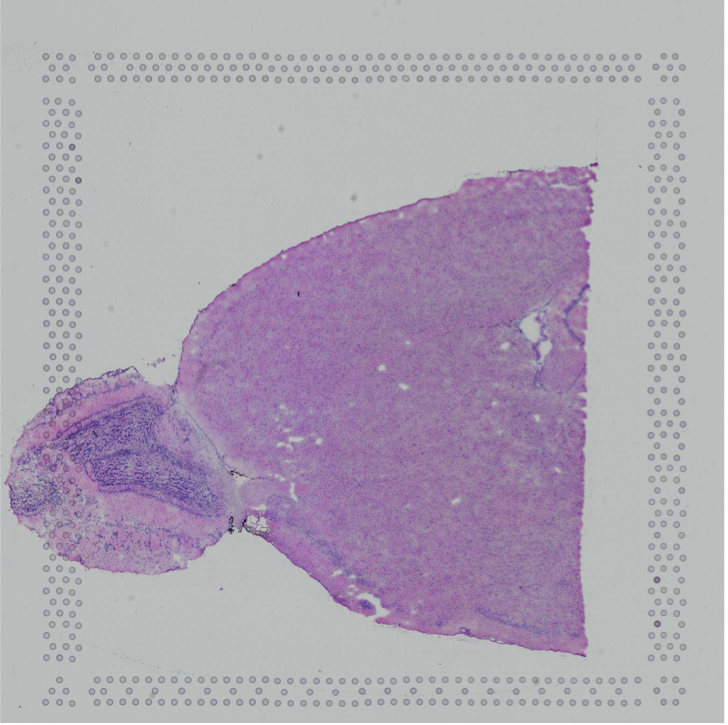


**Steps:**

1. Thresholding:


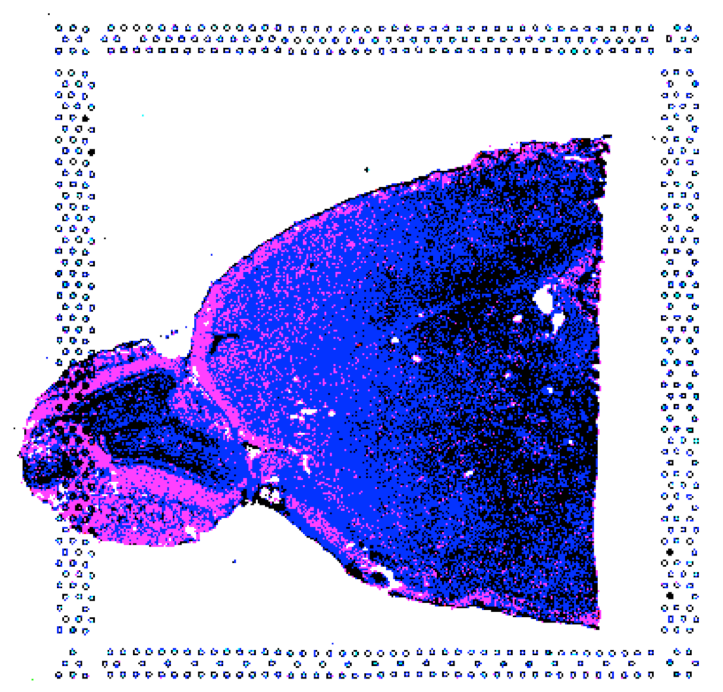


All color values below a specified threshold are set to 0 and all above to 1. The threshold will be computed automatically using k-means (i.e.., using a variant of Otsu's method).

1. Isoblur:


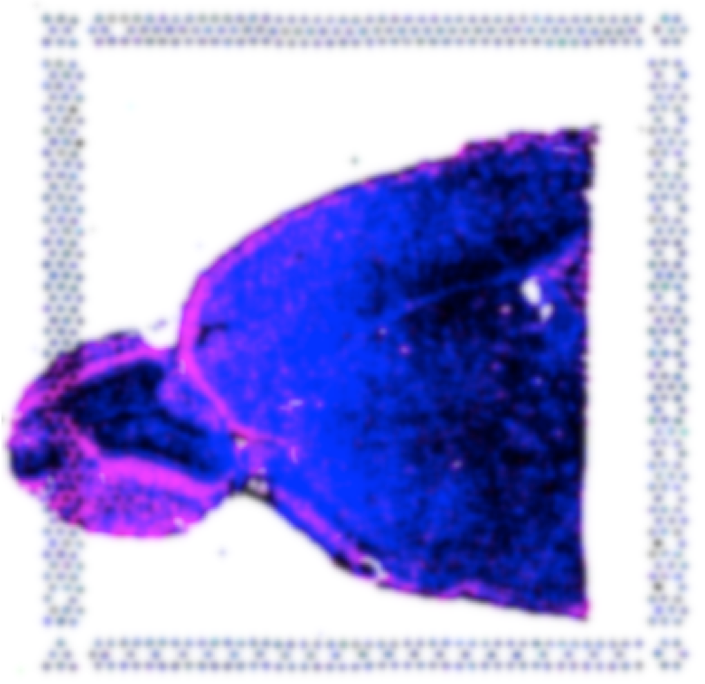


Next, an isotropic blurring filter is applied with a sigma value of 2 to smooth out dust and speckles on the array.

1. Conversion into “superpixels” using the SLIC algorithm:


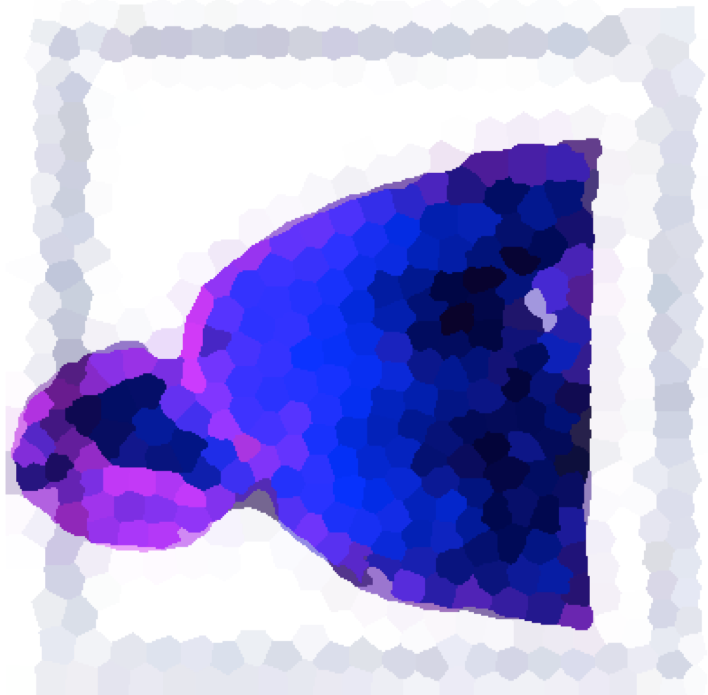


A “superpixel” can be defined as a group of pixels that share common characteristics. Concretely, the SLIC algorithm generates these superpixels by considering both the color similarity (in CIELAB space) and spatial proximity of the constituent pixels.

1. Conversion to CIELAB color space:


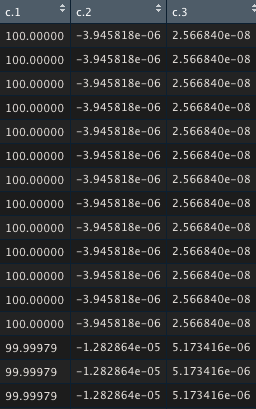


The superpixels are converted into a data.frame with an entry (row) for each pixel. The columns represent the L*a*b channels in the CIELAB color space.

1. K-means clustering:


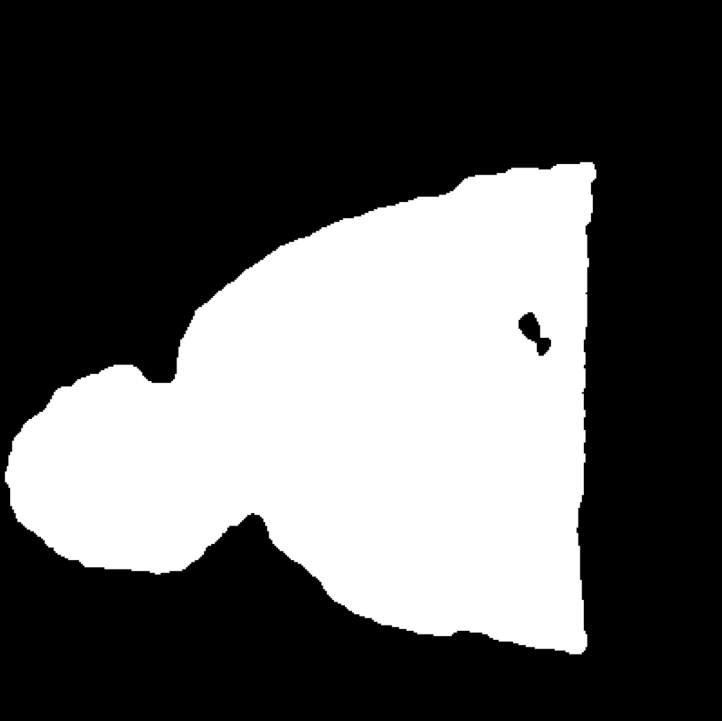


The data.frame with CIELAB colors is clustered using k-means (k = 2) which puts the tissue region and outside region into separate groups. The groups are converted back to an image representation with the values 0 or 1.

1. Median blur:


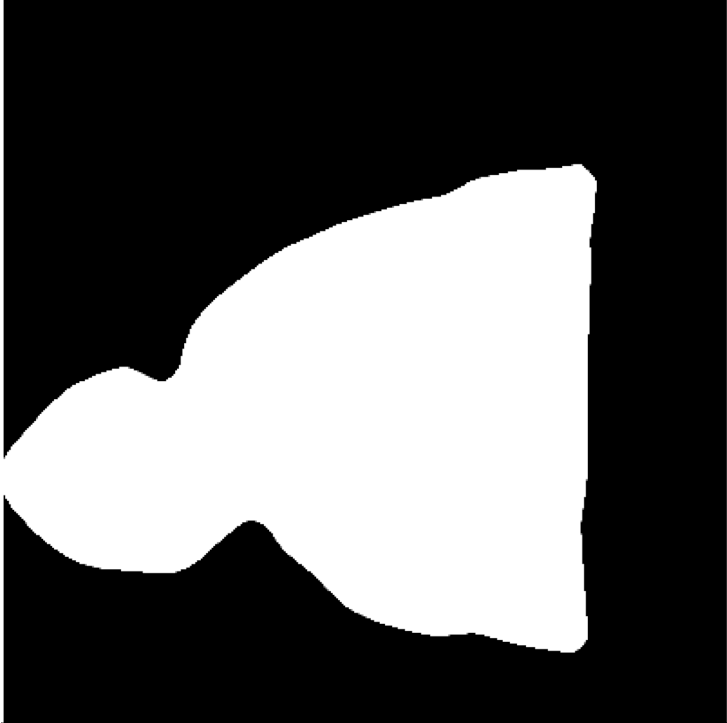


A median filter with a window size of 20x20 pixels is applied to smooth out the regions.

1. Thresholding:


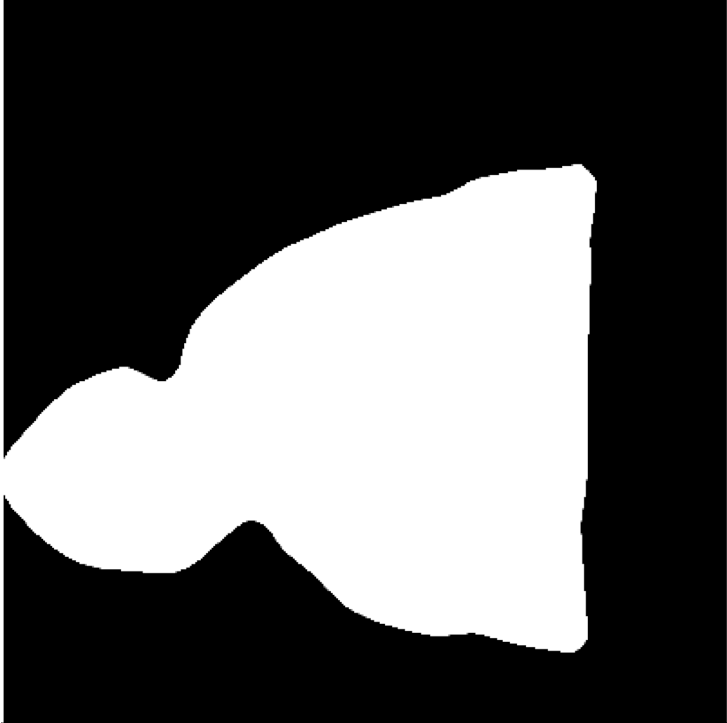


The same threshold as in step 1 is applied to the image again to create the final mask.

1. Choose shapes:


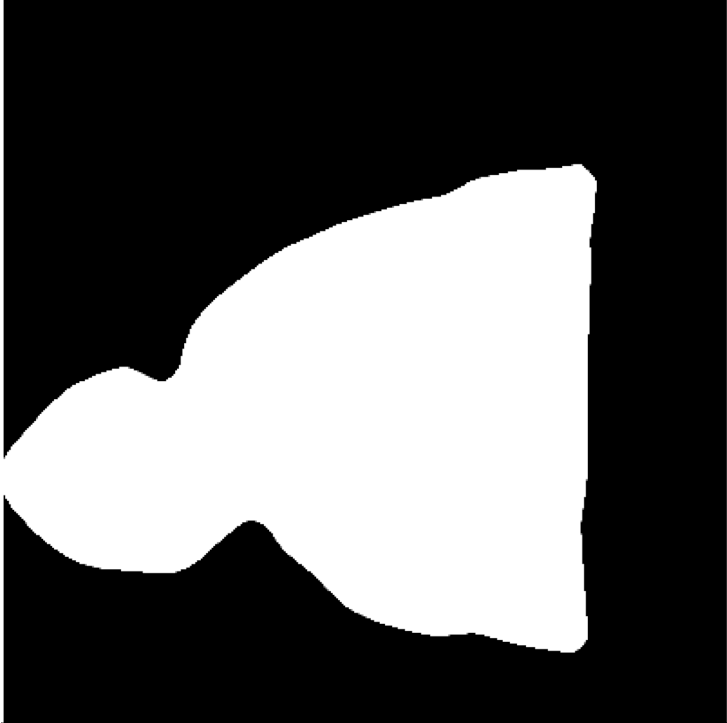


At this point there could be multiple disconnected regions in the mask and either the tissue region or outside region could have a value of 0 or 1. Accordingly, the already established “spots under tissue” (info obtained from the 10x Space Ranger output) is used to determine which area to return.

1. Return mask
