# Supplementary material for: Seamless integration of image and molecular analysis for spatial transcriptomics workflows
Source: BMC Genomics. 2020 Jul 16;21:482. doi: 10.1186/s12864-020-06832-3 (PMC7386244; doi:10.1186/s12864-020-06832-3)
Supplement: Supplementary file 2 — Additional file 2: Supplementary figure S1-S10. [file 12864_2020_6832_MOESM2_ESM.docx]

**Supplementary figures**


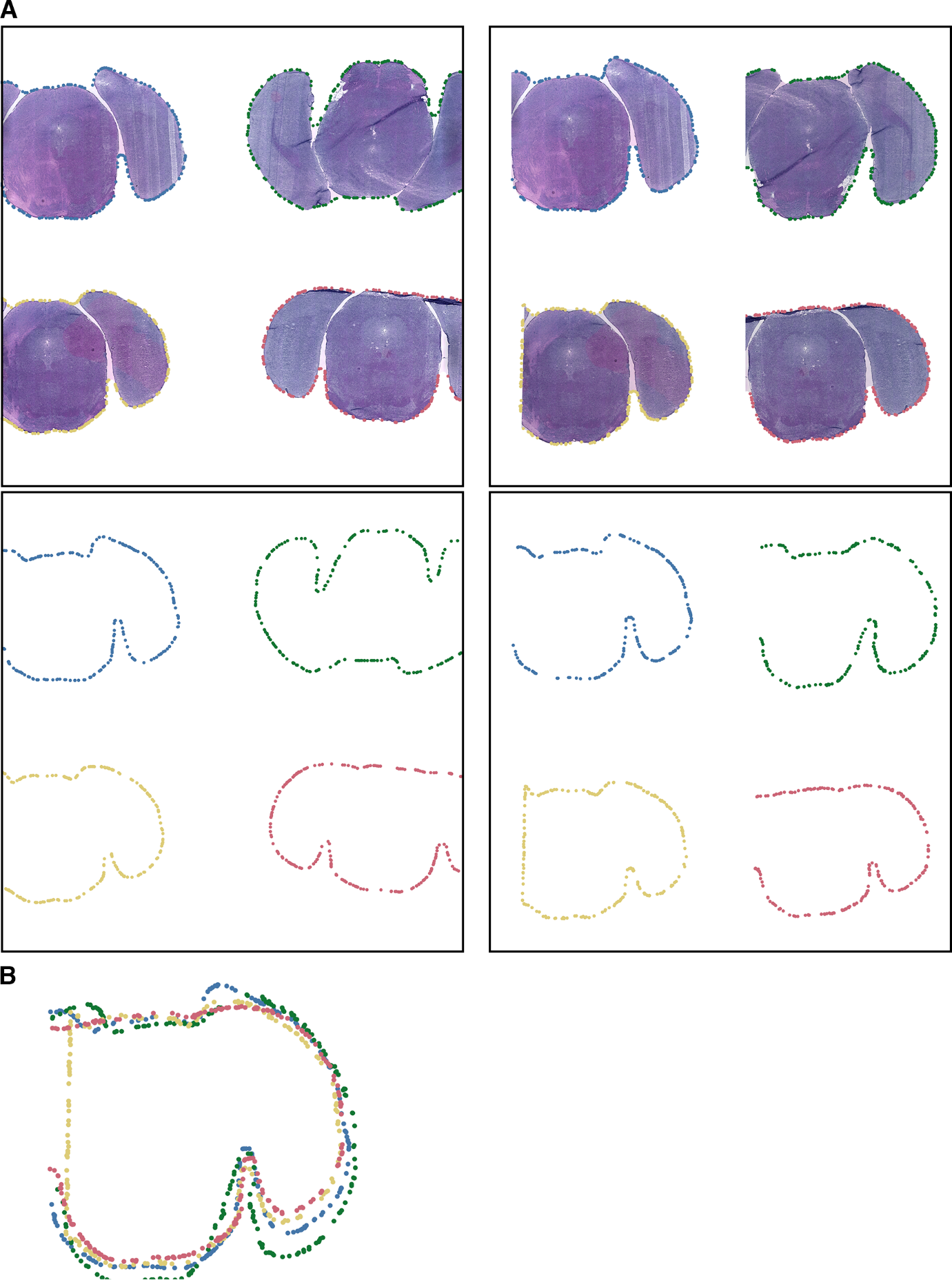


**Figure S1**. **Edge detection and automatic image alignment.** A) A tissue section is selected as the reference image (here section 1 marked with blue dots in the top left corner of each panel) which the other sections are aligned to by an iterative closest point (ICP) algorithm. The left panels show the masked HE images and edges before alignment with a unique color for each section edge. The right panels show the HE images and edges after alignment. As demonstrated, the automatic alignment algorithm can find rotations (sample 2, dark green edges) and reflections (sample 4, red edges) as well as translations which is applied to all three examples. B) With the transformation matrices known, any position can be mapped between the samples and the reference coordinate systems. Here, the aligned edges are overlaid on top of each other to demonstrate the optimal solution found by the ICP algorithm.

**
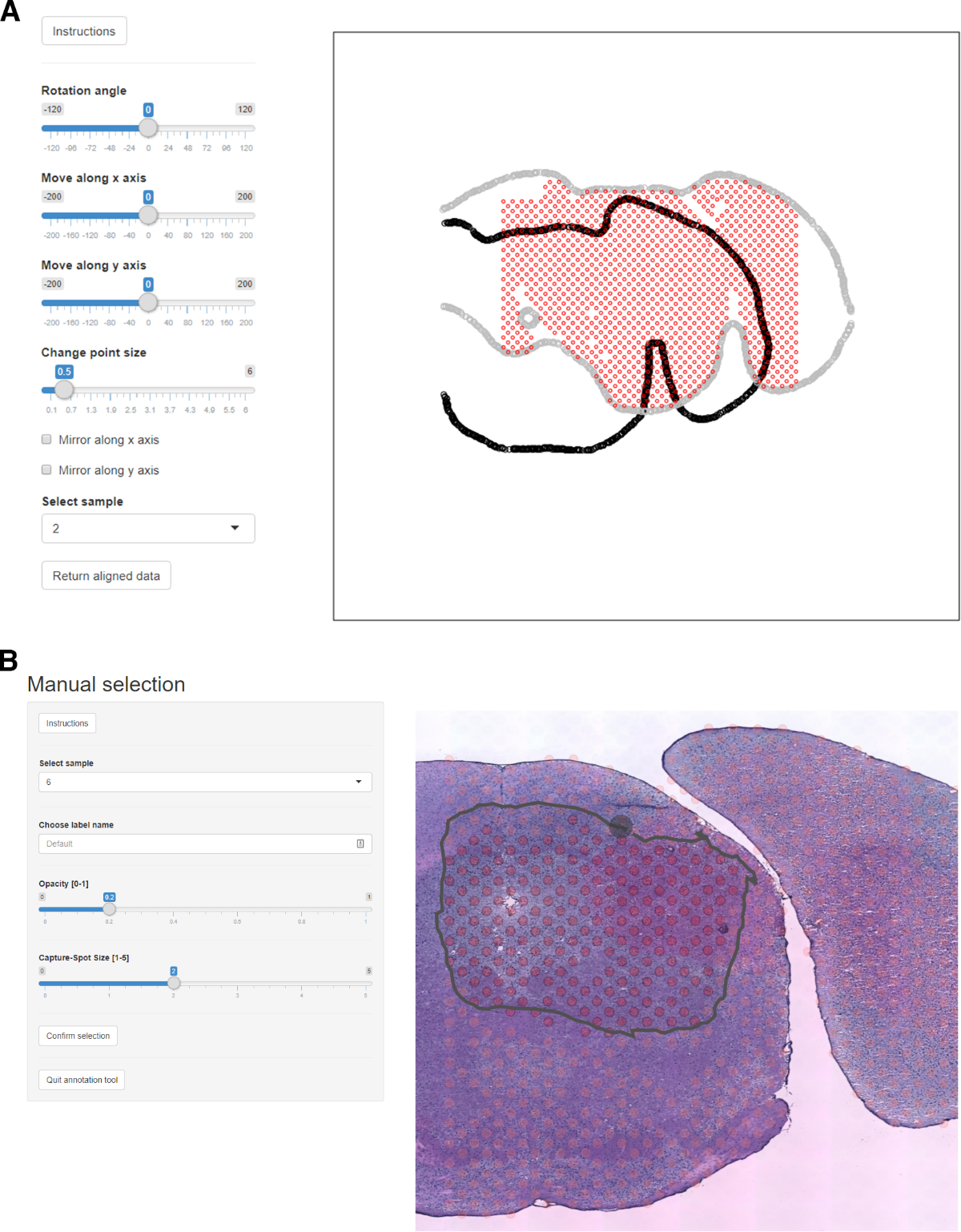
**

**Figure S2. Manual image alignment and annotation**. A) If the automatic alignment does not produce a satisfying result, e.g., due to obstructions of the tissue image, the user can choose to manually align the images. The reference tissue edge is outlined in black and the selected sample edge is outlined in light gray with the associated spots colored in red. B) Each spot can be given a label manually using an interactive selection tool that, for example, can be used to extract capture-spots from certain histological regions to conduct differential gene expression analysis.


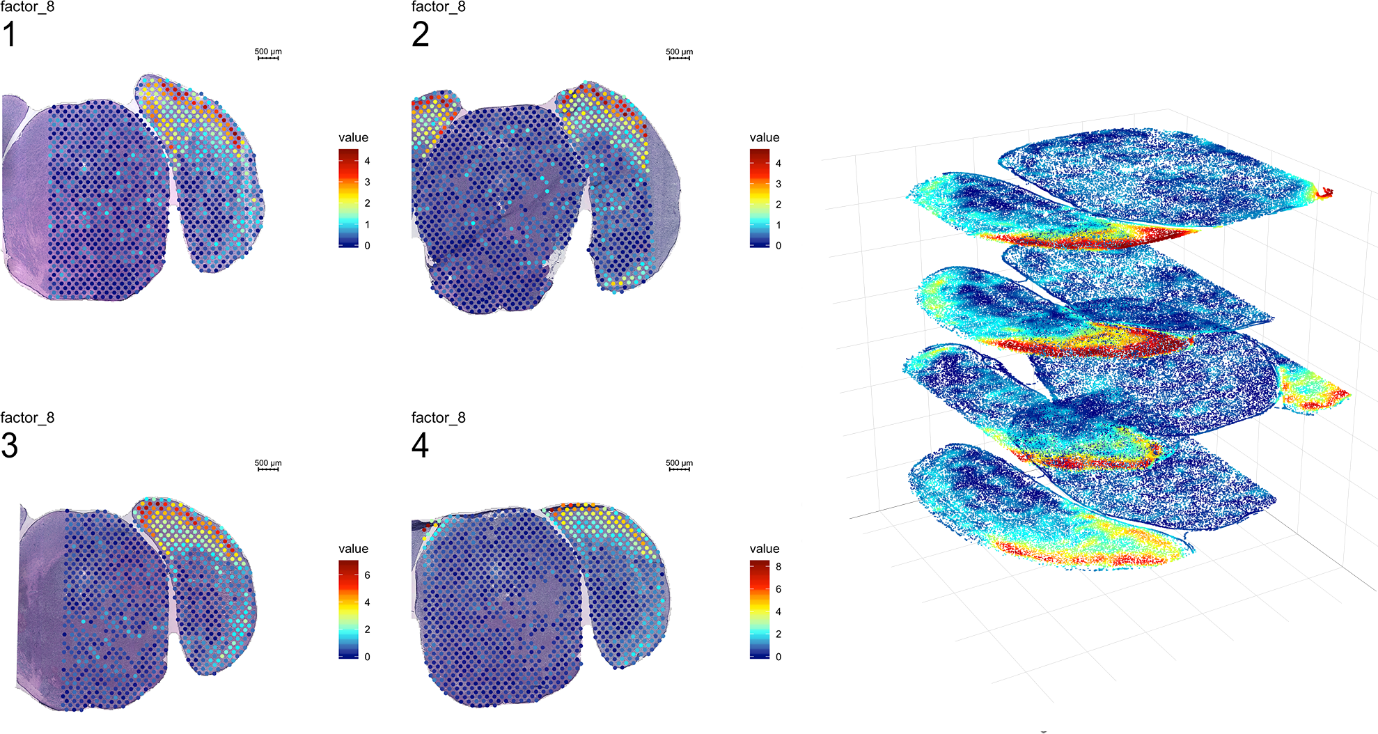


**Figure S3. 3D stack visualization.** The user can interactively turn the image to gain a better perception of the gradually shifting factor and gene activities. The figure shows one of the factors with diminishing activity further posterior in the mouse brain both in 2D for each section separately (left) and in 3D with the sections stacked on top of each other (right).


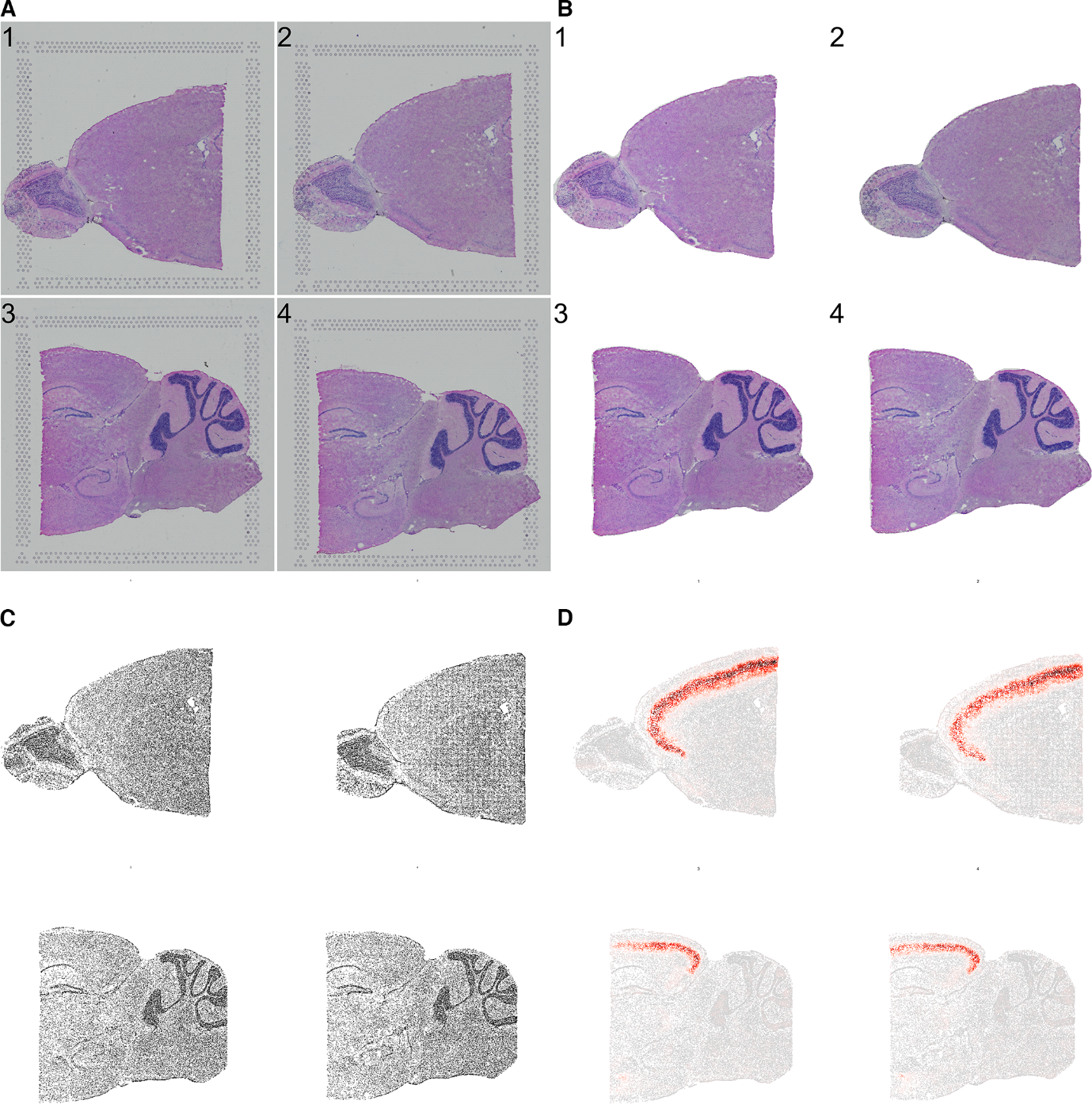


**Figure S4. Point cloud creation and interpolation.** A-B) The raw images are masked to remove the background of the array. C) Point clouds are created by intensity-based detection of cell nuclei. D) Each point within the cloud is assigned to a grid, and interpolation of values across the grid for a chosen feature (here exemplified by one of the NMF factors) is then transferred back to the points.


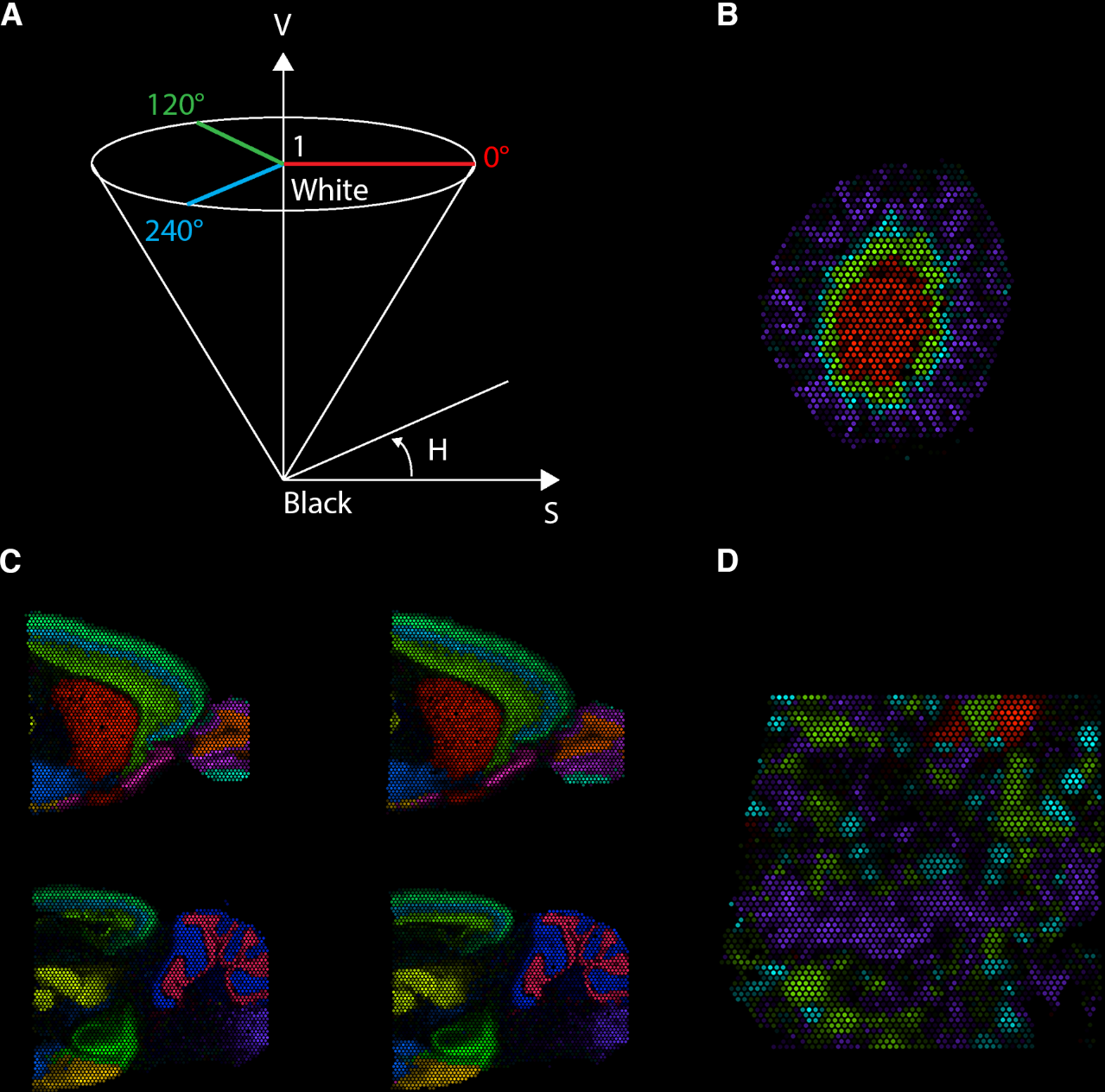


**Figure S5. HSV color scale for multiple feature (e.g. genes, factors) plots. A**) The feature values are rescaled to values between 0 and 1 and encoded in the V-channel. A value of zero gives the capture area a black color regardless of feature, whereas higher values increase the intensity of the corresponding feature color. For each spot, the color is defined by the feature with the highest scaled value. This allows for simultaneous visualization of multiple non-overlapping features, giving a clear representation of, for example, distinct factor-based transcriptomic profiles of interest, as demonstrated for the *mouse kidney* (B), *mouse brain* (C) and *human lymph node* (D) samples.


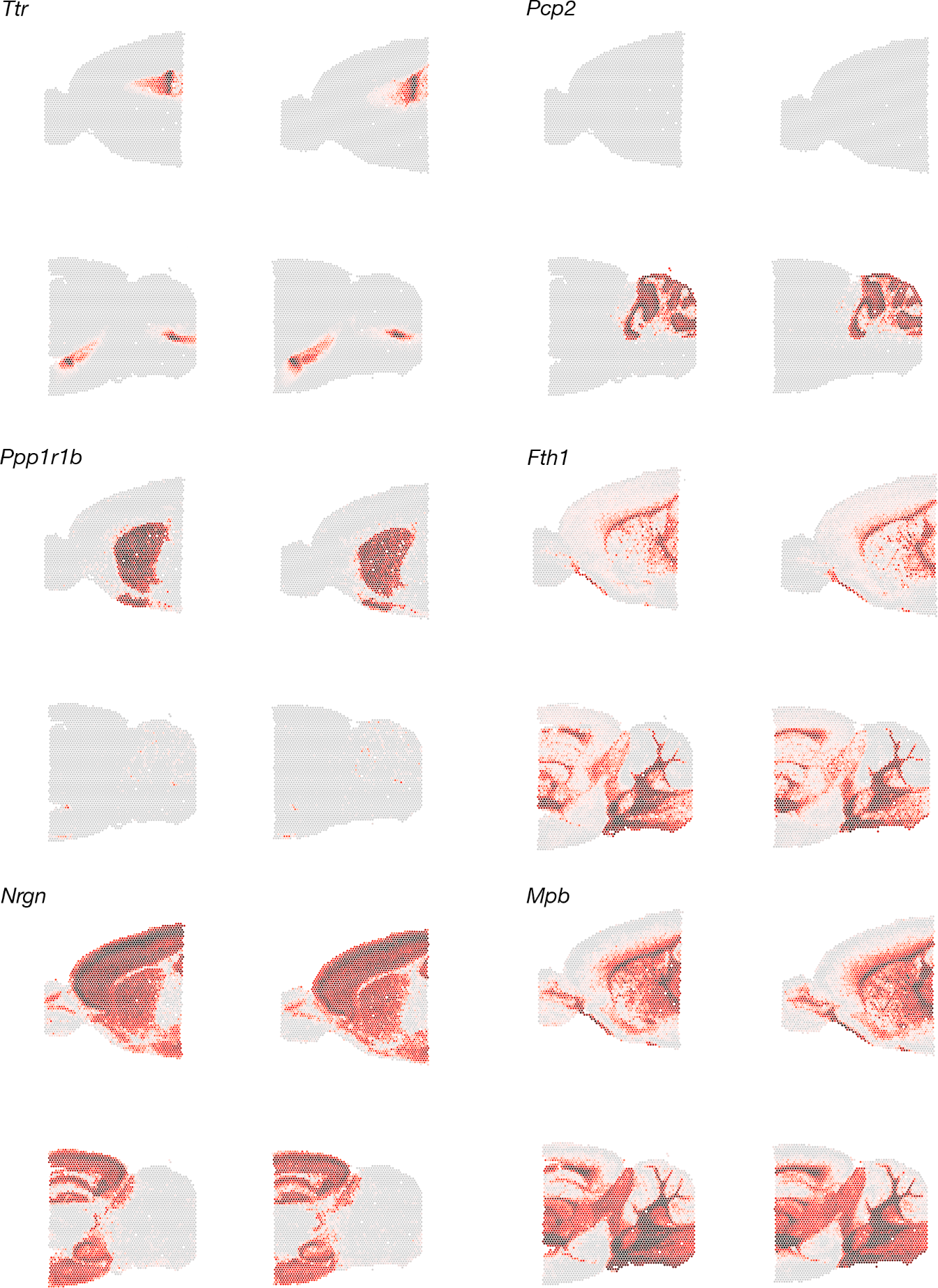


**Figure S6. Spatial autocorrelation.** Genes selected from the top 15 highest rankings based on spatial correlation of transcripts from the mouse brain Visium samples.


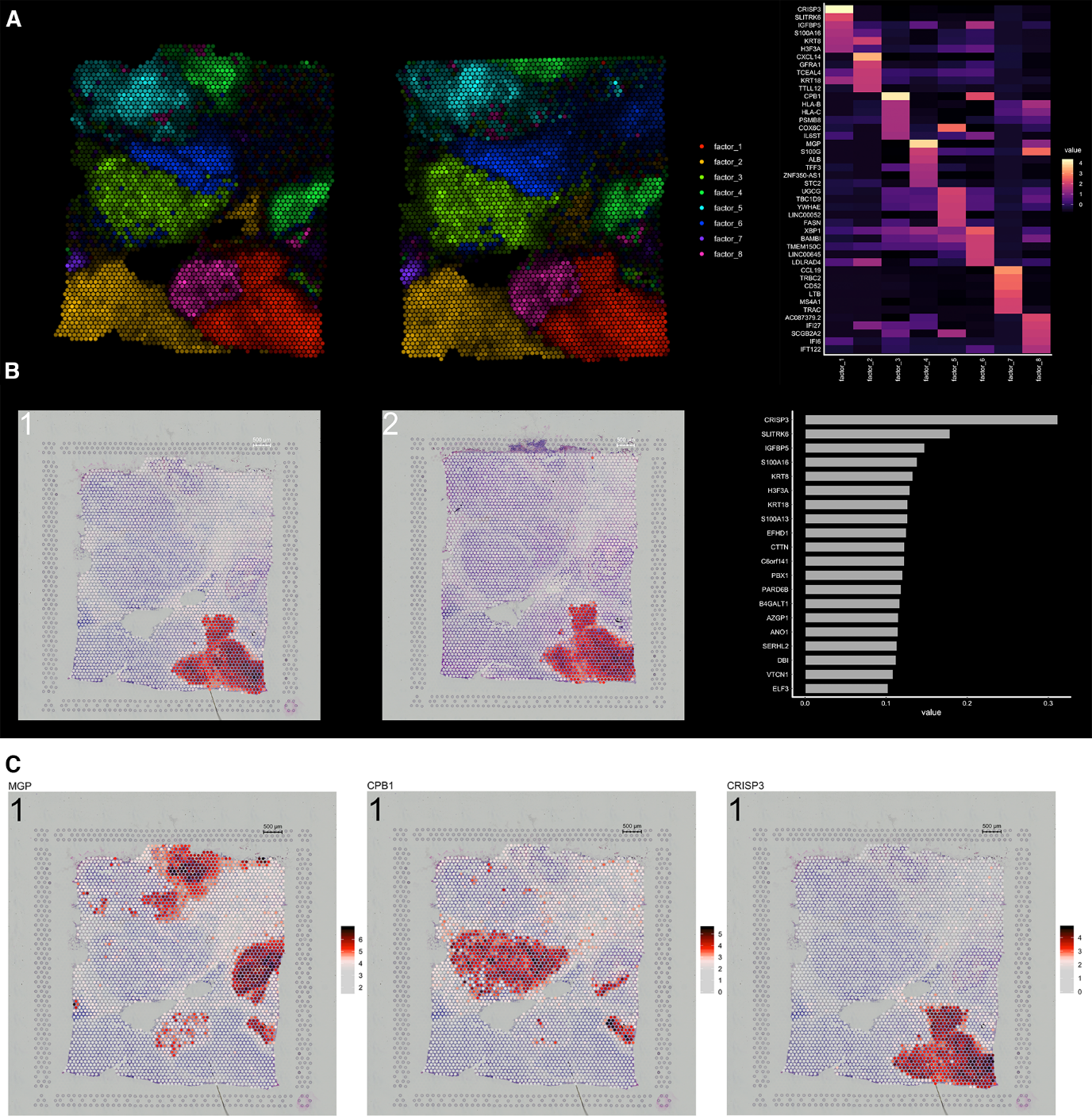


**Figure S7. Breast cancer factor analysis showing spatially confined clusters for the different regions of the tumor with markedly different transcriptomic profiles.** A) HSV color scale plots of the tissue where eight factors are shown simultaneously, factor 1, 2, 3, 4, 5, 8 corresponds to tumor regions. B) Tissue overlay of factor 1 and a histogram of its driver genes. C) Normalized counts plotted for three of the driver genes: MGP (a factor 4 driver gene), CPB1 (a factor 3 driver gene) and CRISP3 (a factor 1 gene).


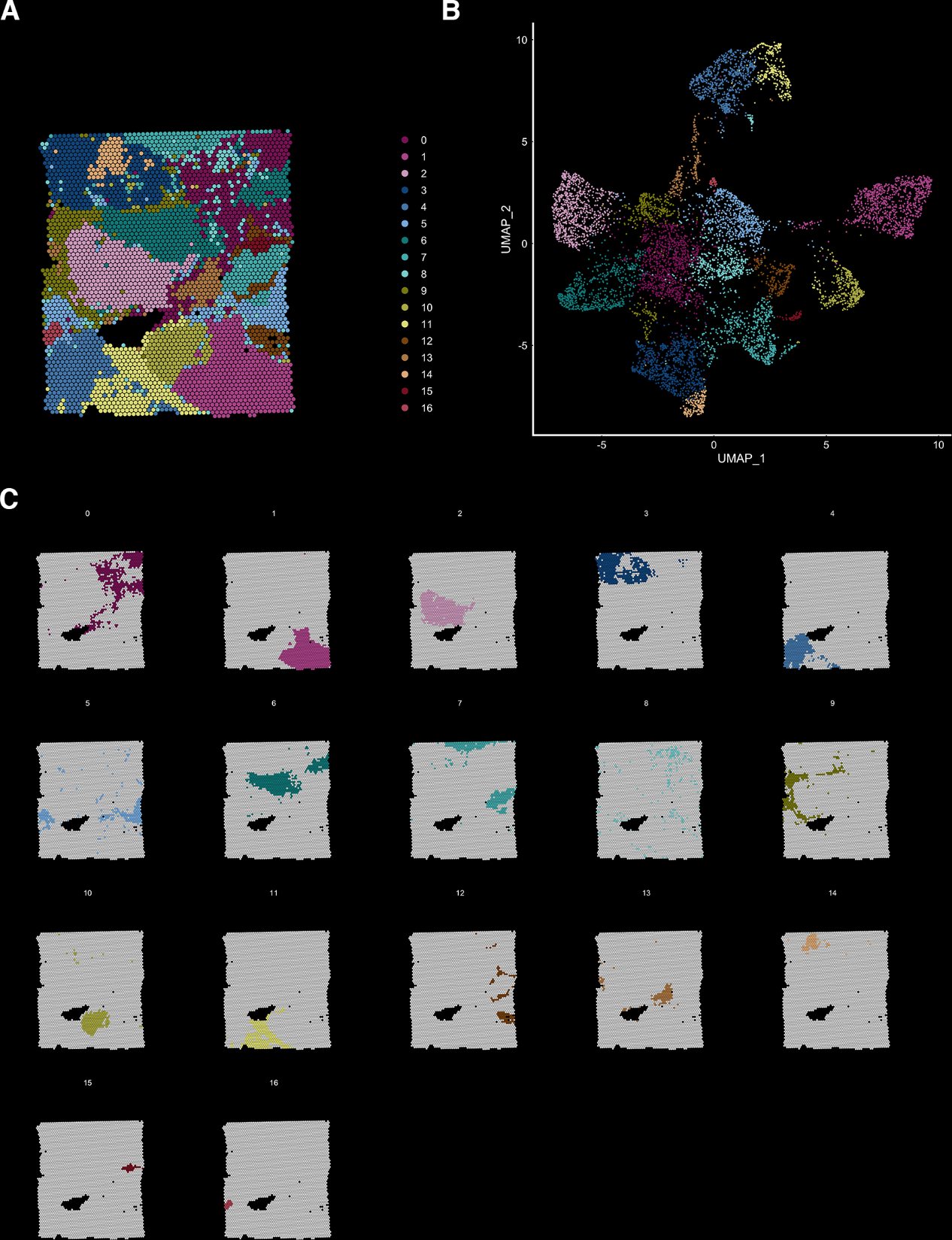


**Figure S8. Breast cancer cluster analysis.** (A) Spatial visualization of Seurat clusters. (B) 2D UMAP embedding of the same clusters as in (A). (C) Cluster split view, showing the spatial locations of each cluster in separate plots.


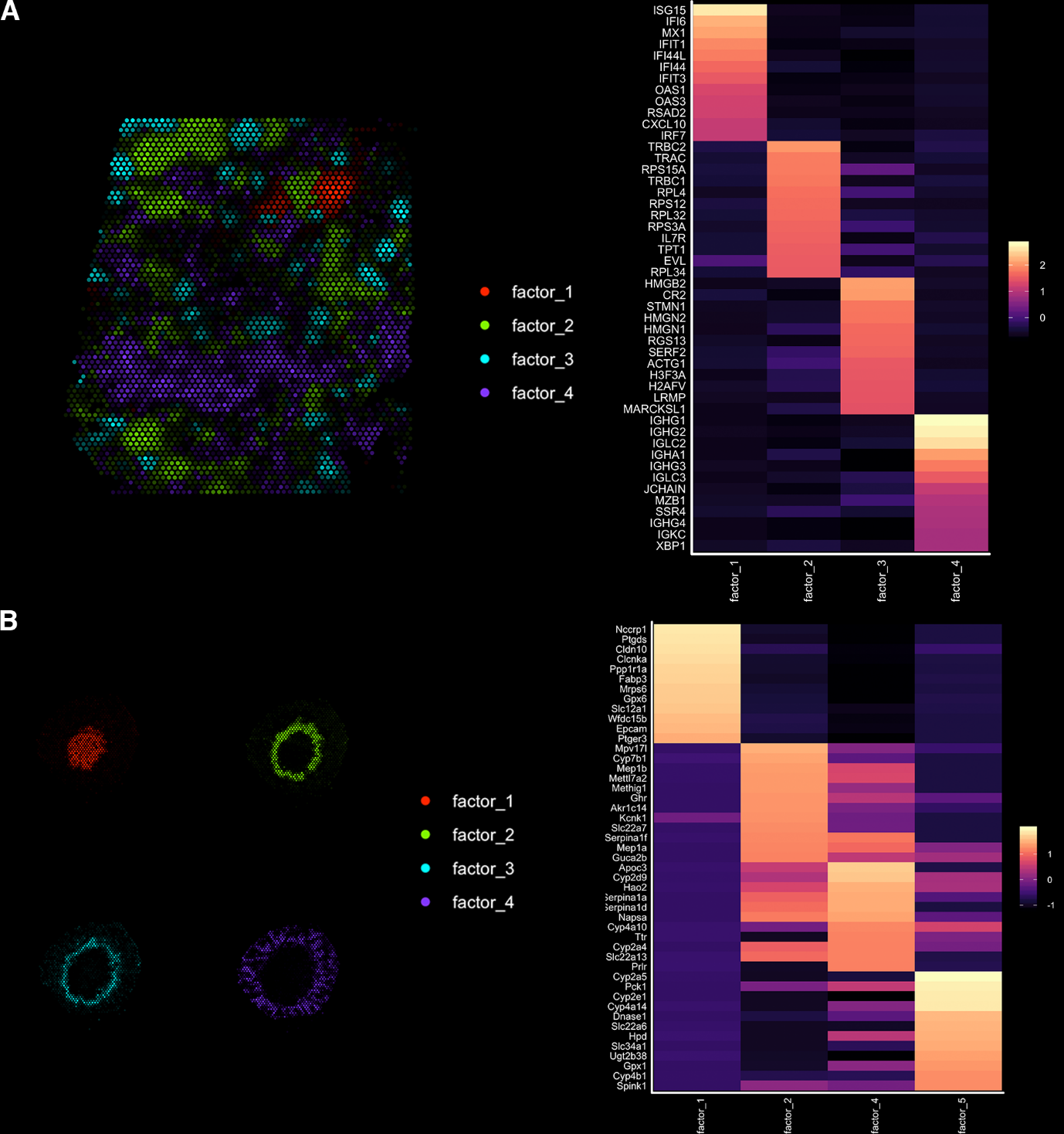


**Figure S9. Factor heatmap and split view of the human lymph node and mouse kidney samples.** A) The lymph node Visium sample is displayed in a HSV color scale plot which shows the spatial position of four of the obtained factors from the NMF and a heatmap of the driver genes for these factors. B) The mouse kidney samples shown in a similar manner as in (A), except that the four factors are visualized with the split feature in STUtility, highlighting each factor individually.


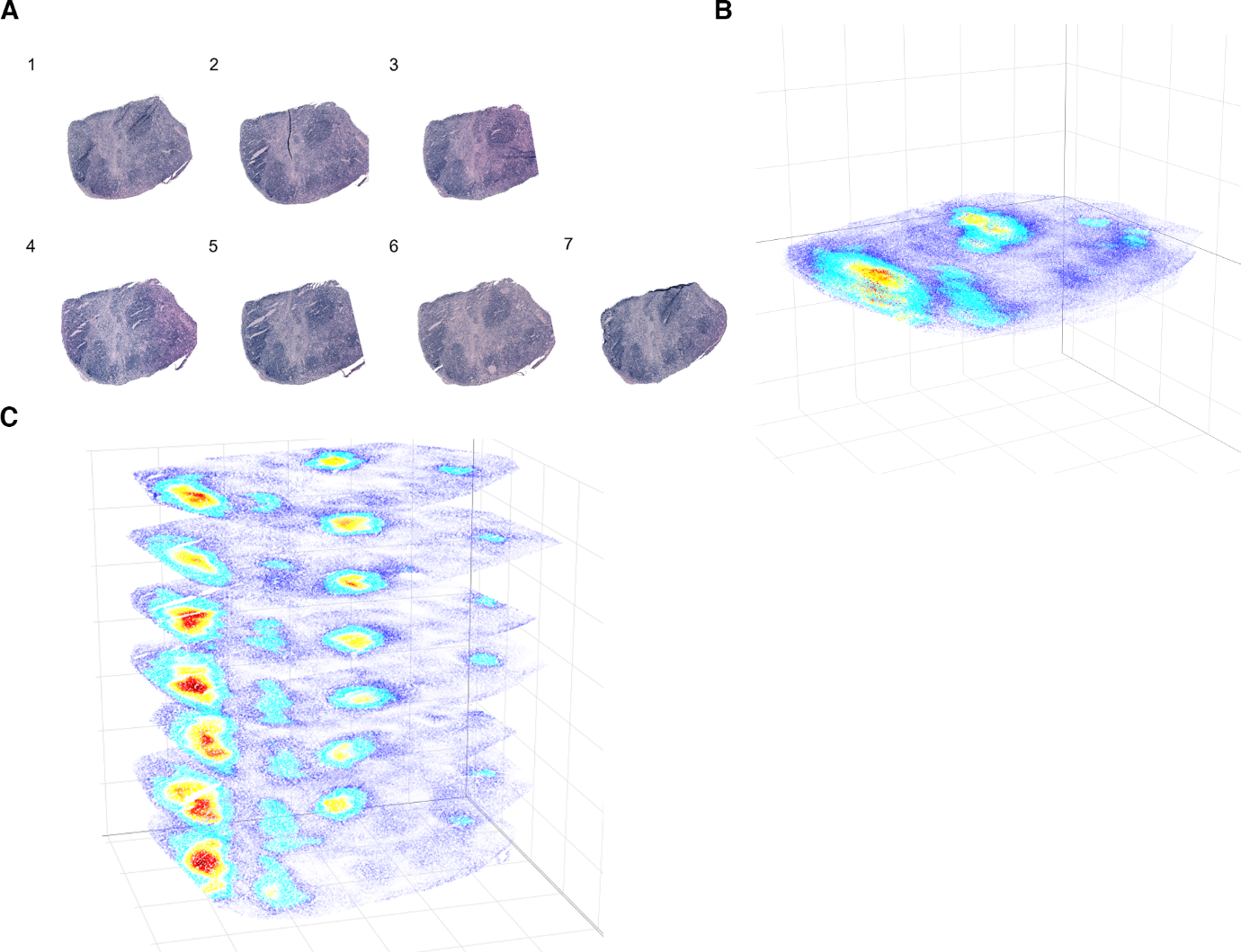


**Figure S10. Visualization of a larger 3D stack of rheumatoid arthritis (RA) samples. A**) Seven consecutive sections were masked and aligned prior to the creation of the 3D stack. B) NMF factor, with driver genes indicative of tertiary lymphoid structure, visualized onto a dense point cloud reflecting actual distance between sections of the original tissue and C) visualized with space between the sections.
