# Supplementary material for: Seamless integration of image and molecular analysis for spatial transcriptomics workflows
Source: BMC Genomics. 2020 Jul 16;21:482. doi: 10.1186/s12864-020-06832-3 (PMC7386244; doi:10.1186/s12864-020-06832-3)
Supplement: Supplementary file 6 — Additional file 6: HTML notebook demonstrating spatial autocorrelation. [file 12864_2020_6832_MOESM6_ESM.html]

 

 

 

 
 
 


 


 spatial autocorrelation 

 
 
 
 
 
 
 
 
 

 
 
 


 


 


 

 

 


 

 


 


 


 spatial autocorrelation 
 Ludvig Larsson 
 6/4/2020 

 


  library(zeallot)
library(ggplot2)
library(STutility)
library(spdep)
library(ggpubr)

load("~/workflowr/STUtility_web_site/pre_data/preSaved_10xHippo_norm_reductions_1_section.RData")  
 
 Spatial autocorrelation 
 
 First we define a modified version of the  CorSpatialGenes  function from STutility to retrun the tablag matrix as well as the scaled gene expression matrix. 
  CorSpatialGenes <- function (
  object,
  assay = NULL,
  slot = 'scale.data',
  features = NULL,
  nNeighbours = NULL,
  maxdist = NULL
) {
  st.object <- GetStaffli(object)

  # Obtain data
  if (is.null(features)) {
    features <- VariableFeatures(object)
  }
  assay <- DefaultAssay(object)
  data.use <- GetAssayData(object, slot = slot, assay = assay)
  data.use <- data.use[features, ]

  # Create a combined network for the samples
  CN <- do.call(rbind, GetSpatNet(object = object, nNeighbours = nNeighbours, maxdist = maxdist))
  resCN <- as.matrix(data.frame(reshape2::dcast(CN, formula = from ~ to, value.var = "distance", fill = 0), row.names = 1))
  resCN[resCN > 0] <- 1
  empty.CN <- matrix(0, nrow = ncol(data.use), ncol = ncol(data.use), dimnames = list(colnames(data.use), colnames(data.use)))
  colnames(resCN) <- gsub(pattern = "\\.", replacement = "-", x = colnames(resCN))
  colnames(resCN) <- gsub(pattern = "^X", replacement = "", x = colnames(resCN))
  empty.CN[rownames(resCN), colnames(resCN)] <- resCN
  listw <- mat2listw(empty.CN)
  fun <- function (x) lag.listw(listw, x, TRUE)

  # Calculate the lag matrix from the network
  tablag <- do.call(rbind, lapply(1:nrow(data.use), function(i) {
    fun(x = data.use[i, ])
  }))
  rownames(tablag) <- rownames(data.use)
  colnames(tablag) <- colnames(data.use)
  
  sp.cor <- unlist(lapply(1:nrow(data.use), function(i) {
    cor(data.use[i, ], tablag[i, ])
  }))

  res <- data.frame(gene = rownames(data.use), cor = sp.cor, stringsAsFactors = F)
  res <- res[order(sp.cor, decreasing = T), ]
  rownames(res) <- res$gene
  
  return(list(data.use, tablag, res))
}  
 If we apply this to our normalized Seurat object, the spatial autocorrelation will be computed on the VariableFeatures defined during SCTransform. We can see how the genes rank based on spatial autocorrelation in the spatgenes data.frame. 
  c(data, tablag, spatgenes) %<-% CorSpatialGenes(se, features = rownames(se@assays$SCT@scale.data))  
 Here I have selected 6 genes with an “autocorrelation” score above 0.8. 
  genes.use <- c("Mbp", "Slc6a3", "Tmsb4x", "Prkcd", "Trh", "Olfm1")
st.object <- GetStaffli(se)

gg <- do.call(rbind, lapply(seq_along(genes.use), function(i) {
  cbind(data.frame(raw_expr = data[genes.use[i], ], 
             tablag_expr = tablag[genes.use[i], ],
             gene = genes.use[i]), st.object@meta.data[, c("pixel_x", "pixel_y", "sample")])
}))

p.list <- lapply(genes.use, function(g) {
  d <- subset(gg, gene %in% g & sample %in% "1")
  p1 <- ggplot() +
    geom_point(data = d, aes(pixel_x, 2000 - pixel_y, color = raw_expr)) +
    facet_wrap(~sample) +
    scale_color_gradientn(colours = rev(RColorBrewer::brewer.pal(n = 11, name = "RdBu")), limits = c(-max(abs(d$raw_expr)), max(abs(d$raw_expr)))) +
    theme_void() +
    theme(strip.text = element_blank(), plot.margin = margin(t = 1, r = 1, b = 0, l = 1, unit = "cm")) +
    labs(title = paste0("scaled gene expression [", g, "]"), color = "")
  p2 <- ggplot() +
    geom_point(data = d, aes(pixel_x, 2000 - pixel_y, color = tablag_expr)) +
    facet_wrap(~sample) +
    scale_color_gradientn(colours = rev(RColorBrewer::brewer.pal(n = 11, name = "RdBu")), limits = c(-max(abs(d$tablag_expr)), max(abs(d$tablag_expr)))) +
    theme_void() +
    theme(strip.text = element_blank(), plot.margin = margin(t = 1, r = 1, b = 0, l = 1, unit = "cm")) +
    labs(title = paste0("spatial lag of scaled gene expression [", g, "]"), color = "")
  p3 <- ggplot() +
    geom_point(data = d, aes(raw_expr, tablag_expr)) +
    geom_smooth(data = d, aes(raw_expr, tablag_expr), method = lm) +
    stat_cor(data = d, aes(raw_expr, tablag_expr)) +
    theme_classic() +
    theme(plot.margin = margin(t = 1, r = 1, b = 0, l = 1, unit = "cm")) +
    labs(x = "scaled gene expression", y = "spatial lag of scaled gene expression", title = paste0("correlation between scaled gene expression \nand spatial lag of scaled gene expression [", g, "]"))
  cowplot::plot_grid(p1, p2, p3, ncol = 3, rel_widths = c(1, 1, 1.2))
})  
  ## `geom_smooth()` using formula 'y ~ x'
## `geom_smooth()` using formula 'y ~ x'
## `geom_smooth()` using formula 'y ~ x'
## `geom_smooth()` using formula 'y ~ x'
## `geom_smooth()` using formula 'y ~ x'
## `geom_smooth()` using formula 'y ~ x'  
  cowplot::plot_grid(plotlist = p.list, ncol = 1)  
   
 And also 6 genes with a lower “autocorrelation” score. Blood related genes are typically exampled of genes with low spatial autocorrelation because the blood vessels do not always form a clear spatial pattern. The other examples include housekeeping genes which shoulld be more or less evenlly distributed across celltypes in the tissue. 
  genes.use <- c("Hbb-bs", "Hba-a1", "Actb", "Tbp", "Rpl31", "Rpl12")
st.object <- GetStaffli(se)

gg <- do.call(rbind, lapply(seq_along(genes.use), function(i) {
  cbind(data.frame(raw_expr = data[genes.use[i], ], 
             tablag_expr = tablag[genes.use[i], ],
             gene = genes.use[i]), st.object@meta.data[, c("pixel_x", "pixel_y", "sample")])
}))

p.list <- lapply(genes.use, function(g) {
  d <- subset(gg, gene %in% g & sample %in% "1")
  p1 <- ggplot() +
    geom_point(data = d, aes(pixel_x, 2000 - pixel_y, color = raw_expr)) +
    facet_wrap(~sample) +
    scale_color_gradientn(colours = rev(RColorBrewer::brewer.pal(n = 11, name = "RdBu")), limits = c(-max(abs(d$raw_expr)), max(abs(d$raw_expr)))) +
    theme_void() +
    theme(strip.text = element_blank(), plot.margin = margin(t = 1, r = 1, b = 0, l = 1, unit = "cm")) +
    labs(title = paste0("scaled gene expression [", g, "]"), color = "")
  p2 <- ggplot() +
    geom_point(data = d, aes(pixel_x, 2000 - pixel_y, color = tablag_expr)) +
    facet_wrap(~sample) +
    scale_color_gradientn(colours = rev(RColorBrewer::brewer.pal(n = 11, name = "RdBu")), limits = c(-max(abs(d$tablag_expr)), max(abs(d$tablag_expr)))) +
    theme_void() +
    theme(strip.text = element_blank(), plot.margin = margin(t = 1, r = 1, b = 0, l = 1, unit = "cm")) +
    labs(title = paste0("spatial lag of scaled gene expression [", g, "]"), color = "")
  p3 <- ggplot() +
    geom_point(data = d, aes(raw_expr, tablag_expr)) +
    geom_smooth(data = d, aes(raw_expr, tablag_expr), method = lm) +
    stat_cor(data = d, aes(raw_expr, tablag_expr)) +
    theme_classic() +
    theme(plot.margin = margin(t = 1, r = 1, b = 0, l = 1, unit = "cm")) +
    labs(x = "scaled gene expression", y = "spatial lag of scaled gene expression", title = paste0("correlation between scaled gene expression \nand spatial lag of scaled gene expression [", g, "]"))
  cowplot::plot_grid(p1, p2, p3, ncol = 3, rel_widths = c(1, 1, 1.2))
})  
  ## `geom_smooth()` using formula 'y ~ x'
## `geom_smooth()` using formula 'y ~ x'
## `geom_smooth()` using formula 'y ~ x'
## `geom_smooth()` using formula 'y ~ x'
## `geom_smooth()` using formula 'y ~ x'
## `geom_smooth()` using formula 'y ~ x'  
  cowplot::plot_grid(plotlist = p.list, ncol = 1)  
   
 
 
 Date 
  date()  
  ## [1] "Thu Jun  4 10:34:55 2020"  
 
 
 Session Info 
  sessionInfo()  
  ## R version 4.0.0 (2020-04-24)
## Platform: x86_64-apple-darwin17.0 (64-bit)
## Running under: macOS Mojave 10.14.6
## 
## Matrix products: default
## BLAS:   /Library/Frameworks/R.framework/Versions/4.0/Resources/lib/libRblas.dylib
## LAPACK: /Library/Frameworks/R.framework/Versions/4.0/Resources/lib/libRlapack.dylib
## 
## locale:
## [1] en_US.UTF-8/en_US.UTF-8/en_US.UTF-8/C/en_US.UTF-8/en_US.UTF-8
## 
## attached base packages:
## [1] parallel  stats4    stats     graphics  grDevices utils     datasets 
## [8] methods   base     
## 
## other attached packages:
##  [1] ggpubr_0.3.0                spdep_1.1-3                
##  [3] sf_0.9-3                    spData_0.3.5               
##  [5] sp_1.4-1                    STutility_0.1.0            
##  [7] SingleCellExperiment_1.10.1 SummarizedExperiment_1.18.1
##  [9] DelayedArray_0.14.0         matrixStats_0.56.0         
## [11] Biobase_2.48.0              GenomicRanges_1.40.0       
## [13] GenomeInfoDb_1.24.0         IRanges_2.22.1             
## [15] S4Vectors_0.26.0            BiocGenerics_0.34.0        
## [17] Seurat_3.1.5                ggplot2_3.3.0              
## [19] zeallot_0.1.0              
## 
## loaded via a namespace (and not attached):
##   [1] reticulate_1.15         tidyselect_1.0.0        htmlwidgets_1.5.1      
##   [4] grid_4.0.0              Rtsne_0.15              munsell_0.5.0          
##   [7] codetools_0.2-16        ica_1.0-2               units_0.6-6            
##  [10] future_1.17.0           miniUI_0.1.1.1          withr_2.2.0            
##  [13] colorspace_1.4-1        knitr_1.28              uuid_0.1-4             
##  [16] ROCR_1.0-11             ggsignif_0.6.0          tensor_1.5             
##  [19] listenv_0.8.0           labeling_0.3            GenomeInfoDbData_1.2.3 
##  [22] polyclip_1.10-0         farver_2.0.3            generics_0.0.2         
##  [25] coda_0.19-3             LearnBayes_2.15.1       vctrs_0.3.0            
##  [28] xfun_0.13               R6_2.4.1                doParallel_1.0.15      
##  [31] rsvd_1.0.3              Morpho_2.8              ggiraph_0.7.0          
##  [34] manipulateWidget_0.10.1 bitops_1.0-6            spatstat.utils_1.17-0  
##  [37] assertthat_0.2.1        promises_1.1.0          scales_1.1.0           
##  [40] imager_0.42.1           gtable_0.3.0            npsurv_0.4-0.1         
##  [43] globals_0.12.5          bmp_0.3                 goftest_1.2-2          
##  [46] rlang_0.4.6             akima_0.6-2             systemfonts_0.2.1      
##  [49] splines_4.0.0           rstatix_0.5.0           lazyeval_0.2.2         
##  [52] broom_0.5.6             rgl_0.100.54            yaml_2.2.1             
##  [55] reshape2_1.4.4          abind_1.4-5             backports_1.1.6        
##  [58] crosstalk_1.1.0.1       httpuv_1.5.2            tools_4.0.0            
##  [61] ellipsis_0.3.0          raster_3.1-5            RColorBrewer_1.1-2     
##  [64] Rvcg_0.19.1             ggridges_0.5.2          Rcpp_1.0.4.6           
##  [67] plyr_1.8.6              zlibbioc_1.34.0         classInt_0.4-3         
##  [70] purrr_0.3.4             RCurl_1.98-1.2          rpart_4.1-15           
##  [73] dbscan_1.1-5            deldir_0.1-25           pbapply_1.4-2          
##  [76] viridis_0.5.1           cowplot_1.0.0           zoo_1.8-8              
##  [79] haven_2.3.0             ggrepel_0.8.2           cluster_2.1.0          
##  [82] colorRamps_2.3          magrittr_1.5            data.table_1.12.8      
##  [85] magick_2.3              openxlsx_4.1.5          readbitmap_0.1.5       
##  [88] gmodels_2.18.1          lmtest_0.9-37           RANN_2.6.1             
##  [91] fitdistrplus_1.0-14     hms_0.5.3               patchwork_1.0.0        
##  [94] shinyjs_1.1             lsei_1.2-0.1            mime_0.9               
##  [97] evaluate_0.14           xtable_1.8-4            rio_0.5.16             
## [100] jpeg_0.1-8.1            readxl_1.3.1            gridExtra_2.3          
## [103] compiler_4.0.0          tibble_3.0.1            KernSmooth_2.23-17     
## [106] crayon_1.3.4            htmltools_0.4.0         mgcv_1.8-31            
## [109] later_1.0.0             tiff_0.1-5              tidyr_1.0.3            
## [112] expm_0.999-4            DBI_1.1.0               MASS_7.3-51.6          
## [115] boot_1.3-25             car_3.0-8               Matrix_1.2-18          
## [118] gdata_2.18.0            igraph_1.2.5            forcats_0.5.0          
## [121] pkgconfig_2.0.3         foreign_0.8-79          plotly_4.9.2.1         
## [124] xml2_1.3.2              foreach_1.5.0           webshot_0.5.2          
## [127] XVector_0.28.0          stringr_1.4.0           digest_0.6.25          
## [130] sctransform_0.2.1       RcppAnnoy_0.0.16        tsne_0.1-3             
## [133] spatstat.data_1.4-3     cellranger_1.1.0        rmarkdown_2.1          
## [136] leiden_0.3.3            uwot_0.1.8              gdtools_0.2.2          
## [139] curl_4.3                shiny_1.4.0.2           gtools_3.8.2           
## [142] lifecycle_0.2.0         nlme_3.1-147            jsonlite_1.6.1         
## [145] carData_3.0-4           viridisLite_0.3.0       pillar_1.4.4           
## [148] lattice_0.20-41         fastmap_1.0.1           httr_1.4.1             
## [151] survival_3.1-12         glue_1.4.0              zip_2.0.4              
## [154] spatstat_1.63-3         png_0.1-7               iterators_1.0.12       
## [157] class_7.3-17            stringi_1.4.6           dplyr_0.8.5            
## [160] irlba_2.3.3             e1071_1.7-3             future.apply_1.5.0     
## [163] ape_5.3  
 


 

 

 

 

 


 
 

 
 
